# Evaluation of Effective Crosslinking Density for Pseudo-Semi Interpenetrating Polymer Networks Based on Polyether Glycols Mixture by Dynamic Mechanical Analysis

**DOI:** 10.3390/polym15010226

**Published:** 2023-01-01

**Authors:** Yajin Li, Bingbing Sun, Yunfei Liu, Zhengzhong Zhang, Yupeng Shen, Haiyang Wang, Xiaojun Liu, Wuxi Xie

**Affiliations:** Xi’an Modern Chemistry Research Institute, 710065 Xi’an, China

**Keywords:** pseudo-semi IPN, TPE, TSE, elastic modulus, effective crosslinking density

## Abstract

Pseudo-semi interpenetrating polymer networks (pseudo-semi IPNs) are a special example of topological isomerism in macromolecules, which have attracted significant attention in recent years with a high potential in a variety of engineering applications of polymeric materials. In this article, pseudo-semi IPNs were synthetized by sequential polymerization of thermoplastic polymers (TPEs) in the presence of thermosetting elastomer (TSEs) with contents of 10, 20, 30, 40 and 50 wt.% in a vacuum oven at 60 °C for about 72 h. In addition, this article describes a method for researching the elastic modulus, effective crosslinking density and physical crosslinking density of TSEs and pseudo-semi IPNs. The inherent interactions and entanglements of pseudo-semi IPNs were discussed by analyzing the changes in elastic modulus and effective crosslinking density at different temperatures. The results show that after the TPE was added to the TSE matrix as a plastic-reinforced material, the ductility increased from 89.6% to 491%, the effective crosslinking density was increased by 100% at high temperatures and the strength of the material matrix was significantly improved. Two physical events take place in our pseudo-semi IPNs as result of energy dissipation and polymeric chains mobility.

## 1. Introduction

In the manufacture of solid rocket propellants, a polymeric substance is frequently employed as a binder to hold together the fuel and oxidizer compounds of the propellant [1]. All solid ingredients are distributed uniformly in a matrix provided by the polymeric binder [2]. The polymeric networks can provide sufficient mechanical strength to the propellant grains. In other words, the mechanical properties of the propellant mainly depend on the integrity of the polyurethane network structure [3,4].

The preparation and properties of multicomponent polymeric systems (blends, IPNs, gels, composites and nano-composites) are of great practical and academic interest [5]. John Millar was the first to coin the term “interpenetrating polymer network” as early as in 1960 [6]. IPNs are in fact a special class of polymer blends, and the two characteristic features of IPNs that distinguish them from the other types of multiphase polymer systems are as follows: (1) IPNs swell but do not dissolve in solvents and (2) creep and flow are suppressed in IPNs. In semi-IPNs, only one component of the assembly is crosslinked, leaving the other in linear form. They are also called pseudo-semi IPNs. Semi-IPN synthesis results in a substantial change in the motional behavior of all materials, and this is due to the molecular-level interpenetration between multiple polymer chains.

**Figure 1:**
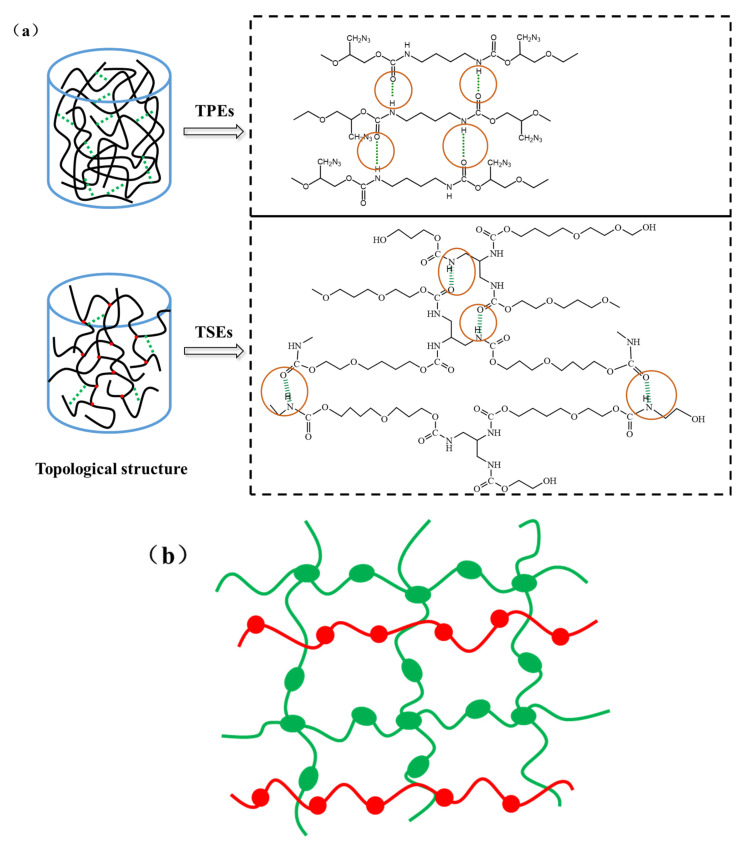
The diagram of network structure for polymers. (**a**) TPEs and TSEs (green imaginary line expresses the intermolecular hydrogen bond (marked by the red circle); red dot expresses the crosslinking point); (**b**) Basic combinations of two polymers for semi-IPN.

Polyurethane elastomer is usually synthesized from hydroxyl-terminated polyether and poly-functional isocyanate (including difunctional isocyanate) [7,8]. At present, including general linear and azides-type polyether binders for the matrix of propellants, we select ethylene oxide/tetrahydrofuran copolyether and azido hydroxyl-terminated prepolymer(glycidyl azide polymer) as a representative polymer in this paper. Their crosslinking structure diagram is shown in Figure 1. For thermoplastic elastomer (TPE) (Figure 1a), the chemical crosslinking effect could be negligible due to the absence of chemical reaction in the system. The physical crosslinking mainly includes inter-chain interaction (Van der Waals force and hydrogen bond), micro area for the gathering of the hard segment and topological entanglement [9,10,11]. Phase separation phenomena between soft and hard segments are responsible for the formation of physical networks.

Van der Waals force is derived from the electrostatic interaction between electrons, as the hydrogen bonds are ideal reversible bonds which easily break and reform due to their lower bond energy than covalent bonds and their dynamic nature [12,13,14]. The hydrogen bond in polyurethane elastomer results from the presence of the –NH group, which is the proton donor, and the carbonyl or ether oxygen groups, which are hydrogen bond acceptors [12,15]. At room temperature, about 80~90% of the –NH groups in the polyurethane hard segment are hydrogen-bonded. Nevertheless, the inadequacies of polyurethane materials are their high temperature performances and chemical resistance that do not to sufficiently meet application demands. In polyether polyurethanes, about 65% of carbonyl groups participate in hydrogen bond formation with the –NH groups, while the ether oxygen presumably accounts for the rest of the –NH associations [16,17]. Coleman et al. has presented a schematic representation of a chain of hydrogen-bonded urethane groups in the amorphous polyurethane [18]. Thus, we introduce strong hydrogen bonds into covalently crosslinked polyurethane, which is named a pseudo-semi interpenetrating polymer network (pseudo-semi IPN) and may increase the energy dissipation and toughness of elastomers.

This paper starts with a preparation of polyurethane elastomers which include thermoplastic elastomer, thermosetting elastomer and pseudo-semi IPNs elastomer, and is followed by an expression of the effective crosslinking density based on the sum of physical crosslinking and chemical crosslinking. Next, the elastic modulus test machine and the measurement method are presented. Finally, the results of the elastomer with effective crosslinking density are discussed and compared with each other. The analysis is based on the dependence of the elastic modulus with temperature.

## 2. Materials and Methods

### 2.1. Materials

The materials used here are commonly used materials for propellants as well as an elastomer with complex nonlinear behavior. Poly(glycidyl azide ether) (*M_n_* = 5500 g/mol) was obtained from Liming Research Institute of Chemical Industry and purified by vacuum drying for 2 h at 80 °C. Isophorone diisocyanate (IPDI, Bayer (Leverkusen city, Germany); *M_n_* = 222.29 g/mol) was used as received. 1, 4-Butanediol (BDO, analytical grade, *M_n_* = 90 g/mol) was obtained from Beijing Chemical Plant and used after it was vacuum-dried for 4 h at 85 °C. Ethylene oxide/tetrahydrofuran copolyether (analytical grade, *M_n_* = 4200 g/mol) was obtained from Hubei Aviation Institute of Chemical Technology and purified by vacuum drying. The catalyst was prepared by the dissolution of dibutyl tin dilaurate (Beijing chemical plant) into diethyl phthalate. Desmodur N100 poly (isocyanate) had an average molecular weight of 728 g/mol and contained 5.3159 mmol/g of –NCO group, which was taken from Beijing Chemical Plant.

### 2.2. Preparation of Polyurethane Elastomer

TPEs: According to our previous reports [19,20], the TPEs and TSEs elastomers are obtained, respectively (the parameters of the samples are shown in Table 1). A general pre-polymerization process was used to prepare poly(glycidyl azide ether)-based TPEs, the hard segments consisted of IPDI chain-extended with BDO. A stoichiometric amount of poly(glycidyl azide ether) was heated and stirred at 90 °C, and then a certain amount of catalyst and IPDI were added. The reaction mixture was stirred and mixed for 2 h at 90 °C. The certain proportional chain extender BDO was added to the above NCO-terminated poly(glycidyl azide ether) prepolymer at 60 °C, and the reaction was kept for 3–5 min. Then, the product was cast in a mold to cure at 100 °C for around 10 h. Finally, the TPEs were obtained.

TSEs: For the TSEs, the preparation method and related performance that were used in this study were as recommended in our previous research [20]. The samples were prepared from poly(isocyanate). The required amount of catalyst was added every time and the mix was stirred, followed by degassing in a vacuum oven at 40 °C. The mixture was finally poured into a Teflon mold and cured under 60 °C for 7 days.

Pseudo-semi IPNs: The pseudo-semi IPNs are prepared by sequential process. The TPEs at different proportions (10, 20, 30, 40 and 50 wt.%) were dissolved in tetrahydrofuran, and the poly(glycidyl azide ether) polymer, curing agent N100 and catalyst were introduced in the elastomer solution. Then, the solvent was slowly evaporated in a vacuum oven at 60 °C around 72 h. For the sake of clarity, the pseudo-semi IPNs samples were named as sample1, sample2, sample3, sample4 and sample5. Some relevant parameters of the samples are summarized in Table 1.

### 2.3. Measurements

The molecular weights of the TPEs were determined on a GPC (LC-20A, Shimadzu, Beijing, Chinacity, country), using THF as the mobile phase at 35 °C with a flow rate of 1 mL/min. The raw data were calibrated using universal calibration with polystyrene standards.

**Figure 2:**
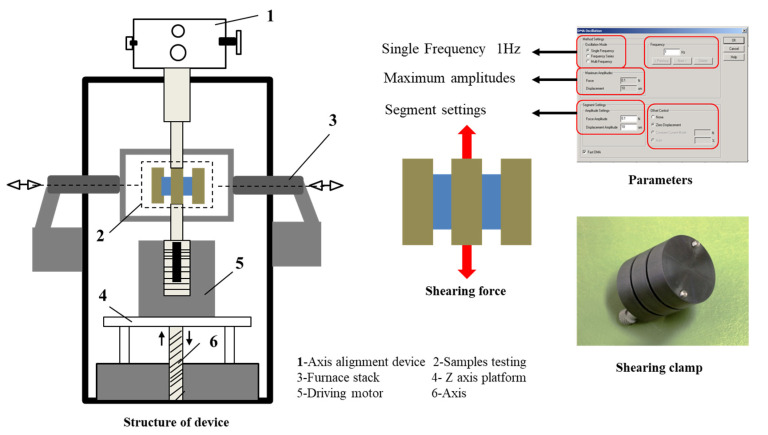
Testing process for the materials.

Temperature dependence of dynamic viscoelastic properties was measured with DMA/SDTA861e at shear model device and a frequency of 1 Hz (Figure 2). The size of the sample used was 4 mm × 4 mm × 2 mm. Measurements were performed in the temperature range from 0 to 100 °C with a heating rate of 3 °C/min under a nitrogen atmosphere. The blocks were longitudinally deformed by a small sinusoidal shear. The variation of the storage modulus (*E*′) as a function of temperature was obtained [21]. The drops in *E*′ curves report on the physical transitions in polymers. The apparent activation energies of the relaxations were determined from the Arrhenius Equation (8).

### 2.4. Methodology

To evaluate the integrity of network structure, Cluff et al. [22] proposed the effective crosslinking density (*V*_e_) of the parameter. Effective crosslinking density is an important parameter in network structure, which is determined with the equilibrium swelling method. Swelling measurements can calculate the degree of crosslinking by assuming the additivity of volumes, using Equations (1)–(5) [23].

(1) $$q_{v}=1+\left(w/w_{0}-1\right)*\left(\rho_{2}/\rho_{1}\right)$$

(2) v2m=1qv

(3) $$\chi_{1}=0.34+\frac{V}{RT}*{\left(\delta_{p}-\delta_{s}\right)}^{2}$$

(4) $$M_{c}=-V\rho *\left(v_{2m}^{\frac{1}{3}}-\frac{v_{2m}}{2}\right)/\left[\ln\left(1-v_{2m}\right)+v_{2m}+\chi_{1}*v_{2m}^{2}\right]$$

(5) $$N_{0}=\rho /M_{c}$$

where *q_v_* is the volume swelling ratio of the elastomer, *w*_0_ is the weight of the specimen before swelling, while *w* is the weight of the specimen after swelling, *ρ*_1_ and *ρ*_2_ are the densities of the solvent and elastomer, *V* is the molar volume of solvent, *δ_p_* and *δ_s_* are the solubility parameters of the polymer and solvent, respectively, and *ρ* is the density of the elastomer, while *N*_0_ is the density of network chain and *Mc* is the apparent average molecular weight.

Based on the equilibrium volume swelling ratios (*q_v_*) of the elastomers, the corresponding volume fractions (*v*_2*m*_) can be calculated by Equation (2). In addition, the Flory–Huggins interaction parameter ($\chi_{1}$) between the elastomer and the solvent can also be evaluated by the Bristow Watson Equation (3). Therefore, the apparent average molecular weight *M_c_* and the density of network chain *N*_0_ can be calculated by Equations (4) and (5), respectively. It is worth mentioning that this method is only suitable for the thermosetting polyurethane elastomer. Additionally, these methods can only help obtain the chemical crosslinking density. With the rapid development of the polymer, researcher prepared various polyurethane elastomers, for example, thermoplastic polymer elastomer which no junction between the molecular chain and interpenetrating polymer network (IPN) which contains a significant amount of topological entanglement. As the effective crosslinking density becomes more and more complicated, the intermolecular forces of the elastomer mainly depend on the physical crosslinking structure. In this case, the equilibrium swelling method is unsuitable for measuring the network structure parameter. The objective of this work is to explore a new method to quantitative calculate the effective crosslinking density of the polyurethane elastomer by measured elastic modulus.

In principle, effective crosslinking density (*V_e_*) is the sum of both chemical and physical crosslinking densities. Additionally, *V_e_* could be expressed by the number of effective network chains per unit volume, as shown in Equation (6). According to the high elastic dynamics theory, Equation (6) can be represented by the following formula in Equation (7), and the effective crosslinking density can be calculated according to the elastic modulus. Based Equation (7), we can obtain the effective crosslinking density under any temperature for the elastomer. In this work, elastic modulus can be obtained by dynamic mechanical analysis.

(6) $$\tau =RT\;*\;V_{e}*\left(\alpha -\frac{1}{\alpha^{2}}\right)$$

(7) $$E^{\prime }=3*V_{e}*RT=3*\left(V_{C}+V_{p}\right)*RT$$

where τ is stress, *V*_*e*_ is the effective chain number per unit volume, which is namely the effective crosslinking density, *V*_*c*_ is the chemical crosslinking density, *V*_*p*_ is the physical crosslinking density, *R* is the general gas constant, *T* is the absolute temperature, *α* is deformation degrees and *E*′ is the measured elastic modulus.

The value of the high elastic modulus is the macroscopic expression of the retraction tension of the macromolecular chain resisting tensile deformation, with the chain attempting to maintain a curly molecular conformation. When the temperature increases, the thermal vibrations of the polymer segments aggravate. Thus, the elastic modulus increases with the rise in temperature.

According to the state equation of rubber, the measured elastic modulus and effective crosslinking density have a dependent relationship with the temperature (Equation (7)). Weisfeld et al. [24] have proposed that the physical crosslinking density of polyurethane elastomer is in agreement with the Arrhenius relationship (Equation (8)), using Equations (7) and (8), we could obtain Equation (9):

(8) $$V_{p}=A*e^{\left(E_{a}/RT\right)}$$

(9) $$E^{\prime }=3*\left(A*e^{\left(E_{a}/RT\right)}\right)*RT$$

Therefore, in order to obtain Equation (10), ln (3*AR*) is in line with 1/*T*, and the *E_a_* and *A* could be obtained by the slope and intercept of the line. Herein, the value of *V*_*p*_ in different temperatures could be calculated by *E_a_* and *A*.

(10) $$\ln\left(E^{\prime }/RT\right)=\ln\left(3AR\right)+\frac{E_{a}}{RT}$$

The physical junctions between molecular chains are formed for mutual entanglement (interpenetrate). In the molecular thermal motion, the physical junction maintains the dynamic equilibrium of disintegration and reconstruction.

For a thermosetting elastomer and pseudo-semi IPN, chemical crosslinking density exhibits no changes in the measurement temperature. Thus, the variation of effective crosslinking density almost comes from the physical crosslinking density, which could be expressed by Equation (11):

(11) $$\left(V_{e,T_{i}}-V_{e,T_{j}}\right)=\left(V_{p,T_{I}}-V_{p,T_{j}}\right)=A*\left(e^{E_{a}/RT_{i}}-e^{E_{a}/RT_{j}}\right)$$

According to Equation (11), the fitting curve of $y=y_{0}+A_{1}\cdot e^{\frac{x}{t_{1}}}$ could be obtained by plotting Δ*V*_*e*_ and 1/*T*. Then, the activation energy and pre-exponential factor of the physical crosslinking dissociation for the material were calculated. By taking advantage of the activation energy and pre-exponential factor, we could obtain the chemical crosslinking density (*V*_*c*_) and physical crosslinking (*V*_*p*_).

## 3. Results and Discussions

### 3.1. Characterization by FTIR

**Figure 3:**
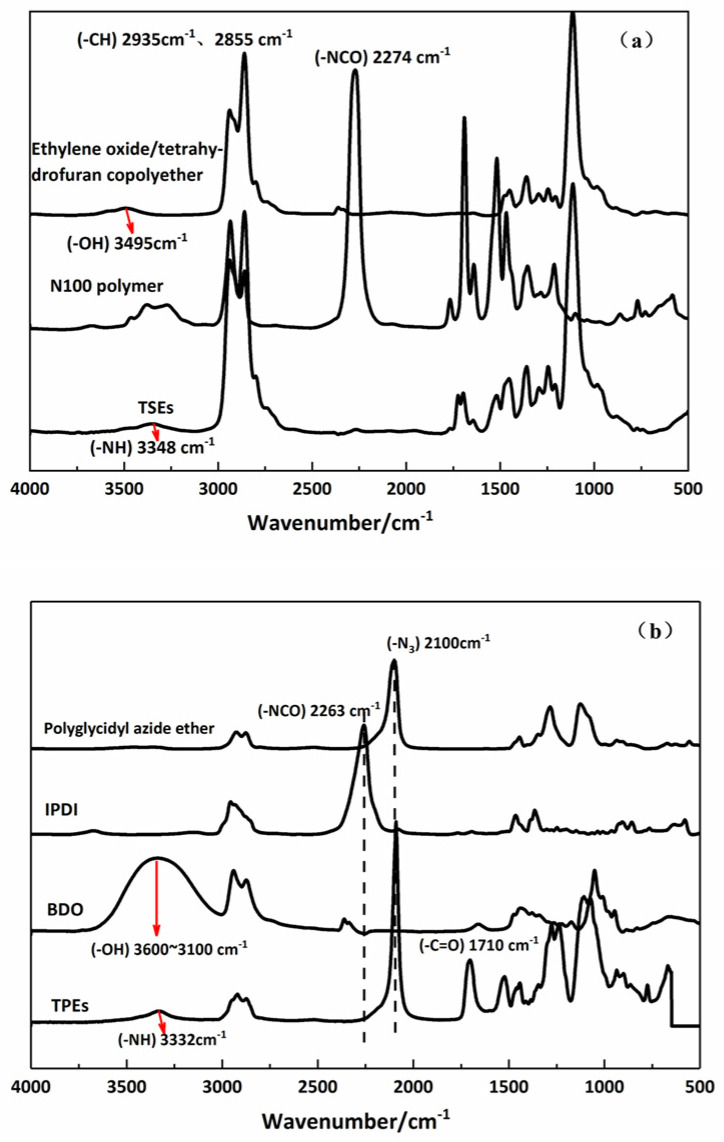
FTIR spectra confirm the forming of TSEs and TPEs. (**a**) TSEs reacting ethylene oxide/tetrahydrofuran co-polyether –OH with N100 –NCO polymer; (**b**) TPEs reacting poly(glycidyl azide ether) –OH with IPDI-NCO compound and BDO –OH compound.

It has been reported that infrared spectral analysis has been widely applied to study microphase segregation in polyurethanes [25,26]. The characterizations of the prepared elastomers are performed by FTIR spectroscopy. Figure 3a shows the IR spectra of the TSE elastomer, ethylene oxide/tetrahydrofuran copolyether and N100. The clear peaks at 2935 cm^−1^ and 2855 cm^−1^ correspond to the C–H vibration for the ethylene oxide/tetrahydrofuran copolyether, with an evident characteristic peak at 3495 cm^−1^ ascribed to the stretching vibration of the O–H group and a C–O–C symmetric stretching vibration appearing at 1130 cm^−1^. In addition, the characteristic absorption peak at 2274 cm^−1^ belongs to the NCO stretching vibration of N100. As seen from Figure 3a, the IR spectra do not exhibit the absorption bands at 3495 and 2274 cm^−1^ corresponding to the –OH and –NCO groups of the TSEs. The urethane characteristic bands at 3348 and 1720 cm^−1^ are also observed; they correspond to the –NH stretching and –C=O stretching of amide. The FTIR spectra of the prepared TPE elastomer and oligomer are shown in Figure 3b. Similarly, the IR spectra do not exhibit the absorption bands at 3495 and 2274 cm^−1^ corresponding to the –OH and –NCO groups. Additionally, the characteristic peaks of the –N_3_ group from the poly(glycidyl azide ether) polymer at 2100 cm^−1^ are still present. The carbonyl groups in the hard segments, which are capable of having hydrogen bonds, and the other carbonyl-containing hard segments exhibited characteristic peaks at lower wave numbers compared to the carbonyl groups with van der Waals interactions. The wave number range of 1690–1710 cm^−1^ (hydrogen bonding) is compared to the carbonyl absorption at the wave number range of 1715–1730 cm^−1^ (free interaction).

**Figure 4:**
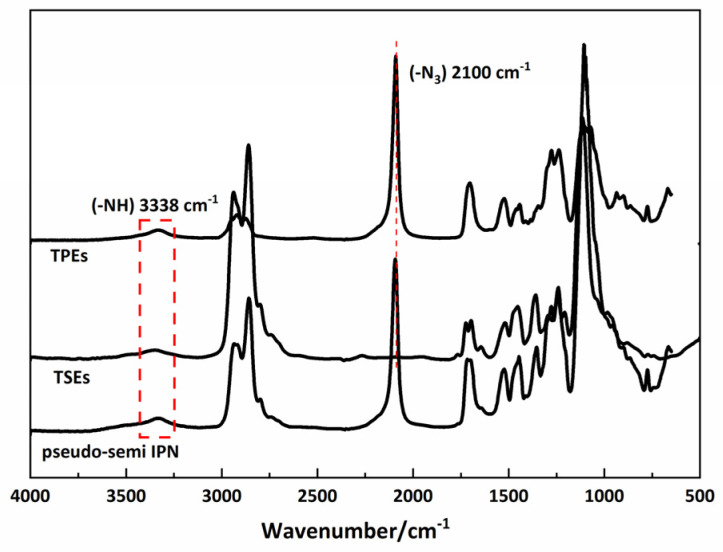
TPEs, TSEs and pseudo-semi IPNs sample FTIR spectra.

Figure 4 shows that the IR spectra of the pseudo-semi IPNs elastomer, the –NH stretching of urethane and the –N_3_ stretching of the TSE polymer have the same peaks, while the hydrogen bonds belonging to carbonyl groups have a lower wave number, giving soft crystalline domains, which indicates that the linear TSE polymer enhances the ability of hydrogen bond formation.

### 3.2. The Effective Crosslinking Density of TPEs

**Figure 5:**
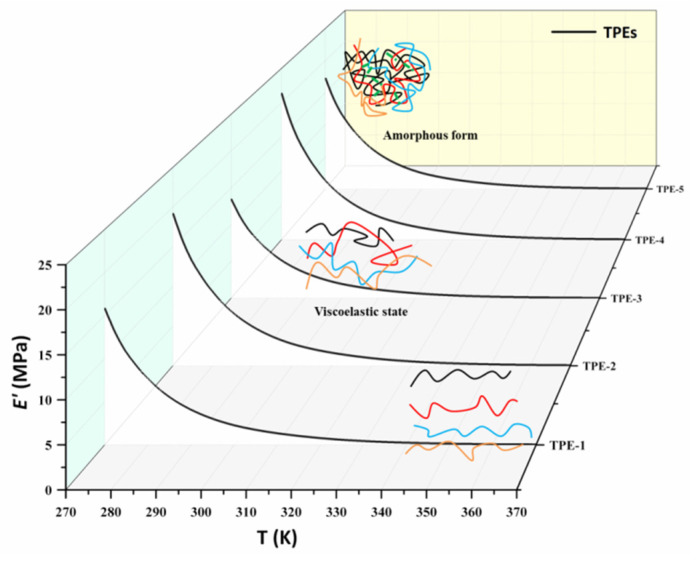
Elastic modulus of TPEs as a function of temperature.

Figure 5 presents the measured elastic modulus (*E*′) as a function of temperature between 273 and 373 K of the TPEs. This temperature range is above the glass transition temperature (*T_g_*) of all samples (Table 1). The curve of *E*′ for the TPEs decrease sharply as the temperature increases from 273 to 373 K, and the values remain steady after 353K. It is understandable that all elastic modulus for TPEs come from the contribution of physical crosslinking, for which the chemical crosslinking effect could be negligible for the thermoplastic elastomer, as the hydrogen bond and physical tangles weaken and diminish following the temperature increases [8]. Lower *E*′ values mean higher mobility.

**Figure 6:**
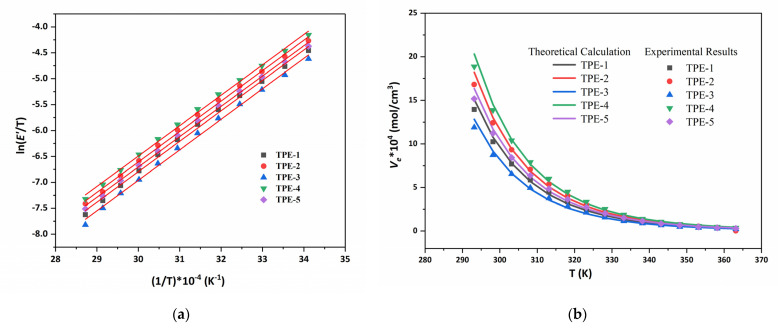
(**a**) Relationships between Δ*V_e_* and 1/*T* of TPEs. Red line is the regression results; (**b**) Effective crosslinking density of TPEs, theoretical calculation and experimental results.

According to the formula Equation (10), the relationship of ln(*E*′/*T*) versus 1/*T* is illustrated in Figure 6a. Table 2 shows the values of *E_a_* and *A* of TPEs calculated from Figure 5 and Equation (11). From that, the physical crosslinking density for different temperatures can be calculated in Figure 6b. Meanwhile, Figure 6b presents the measurements in terms of effective crosslinking density of the TPEs. The points of the experimental measurements and lines are obtained by the formula, as discussed previously. We obtain a good match between the theoretical calculation represented by the line and the measures displayed as points as in Figure 6b.

At low temperatures, the physical crosslinking density in the TPEs is slightly larger than the effective crosslinking density. Afterward, there was a reduction in *E*′ as the temperature increased, which may be due to an increase in polymer segmental motion. The physical crosslinking density decreases with the increase in temperature, which is closer to the effective crosslinking density. These results indicate that the effective crosslinking density of the TPEs is highly dependent on temperature. As we all know, with increasing temperature, complex phase behaviors are exhibited with the polyurethane network formed by hydrogen bonds. This is the reason why, at higher temperatures, the mechanical characteristics are out of applicability.

### 3.3. The Effective Crosslinking Density of Pseudo-Semi IPN

**Figure 7:**
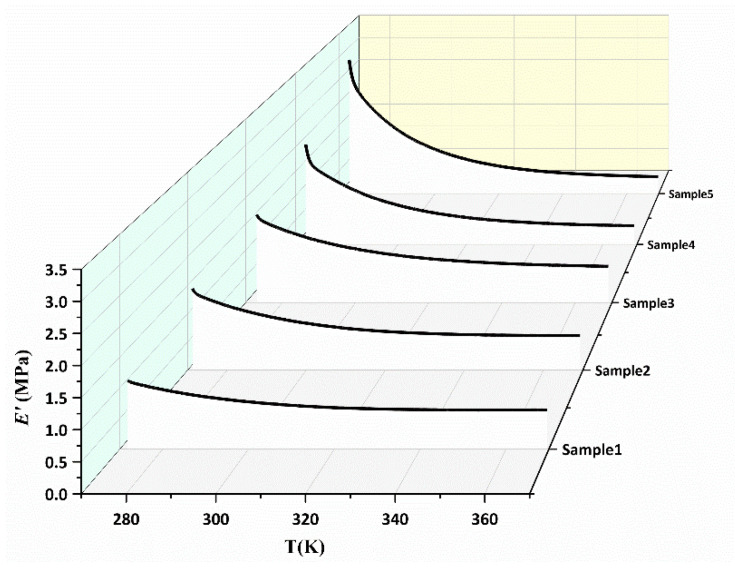
Elastic modulus of pseudo-semi IPNs as a function of temperature.

For pseudo-semi IPN elastomers, the curves of *E*′ exhibit a slow decline as the temperature increases, which includes the increase in conformational entropy and the decrease in physical crosslinks (Figure 7). The interpenetration resists the creep of flow to a greater extent than copolymers or blends. Evidently, the reduction in physical crosslinking in the TPEs system contributes more to the modulus than the increase in conformational entropy. Thus, the *E*′ of pseudo-semi IPNs remains a quantitative value at high temperatures. In view of this, interpenetration may significantly improve many physical properties of the final material relative to any single polymer characteristic. Two physical events take place in our pseudo-semi IPNs as a result of energy dissipation and polymeric chains mobility. The major reason for the difference in the properties between polymer blends and semi-IPNs is the latter greater adhesion and better mixing. The inclusion of linear polyurethane into a crosslinked network puts the micro-phase separation phenomenon into a different context. This mobility may bring reactive parts of the network closer and facilitate the completion of some incomplete crosslinking reactions.

**Figure 8:**
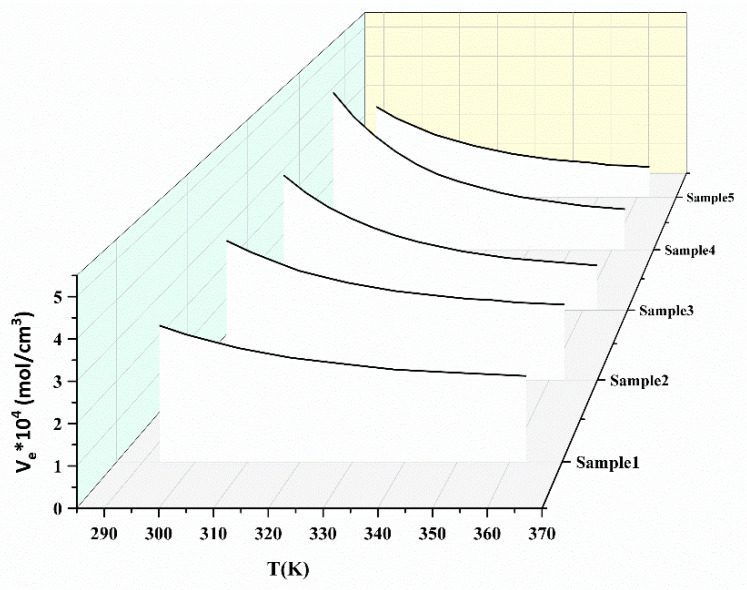
Effective crosslinking density of pseudo-semi IPNs based on Equation (7).

**Figure 9:**
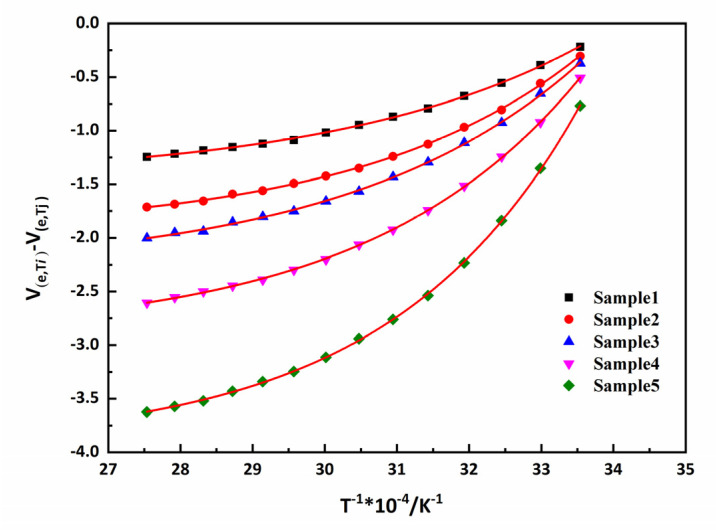
Relationships between Δ*V*_*e*_ and 1/*T* of pseudo-semi IPNs. Red line is the regression results.

According to the formula Equation (7), the effective crosslinking density is illustrated in Figure 8. This figure shows the dependency of the *V*_*e*_ of the networks with temperature in different samples. Meanwhile, the *V*_*e*_ values of the samples exhibited a non-monotonic change with the proportion of TPEs. This is may be due to the different physical crosslinking densities that are formed. Analogously, according to the formula in Equation (11), the relationship of Δ*V*_*e*_ versus 1/*T* is illustrated in Figure 9. The activation energy and pre-exponential factor of the samples could be obtained and the values are given in Table 3.

**Figure 10:**
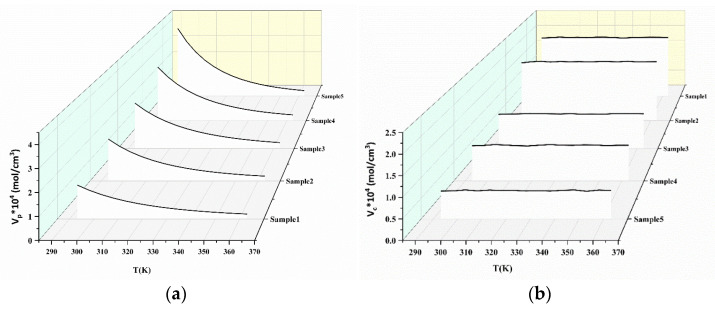
Variation of physical crosslinking density (**a**), chemical crosslinking density (**b**) with temperature for pseudo-semi IPNs.

Based on the Δ*V_e_* and 1/*T* analysis, the physical crosslinking density and chemical crosslinking density of several pseudo-semi IPN samples are shown in Figure 10 along with the temperature. By increasing the temperature, the value of chemical crosslinking density remains basically unchanged for all the samples. Additionally, the data of chemical crosslinking density decrease with the TSE contents. All the transformations are part of the hard segment interval of the TPE that is embedded in the TSE network. A limited gain in mobility after 60 °C is monotonously amplified around 95 °C due to the onset of hydrogen bond dissociation. At a given moment, the TPE chain segments may slip together, but the TSE network confines the flowing to a certain extent. The *V*_*c*_ data confirmed that the segment volume of TSE in the pseudo-semi IPNs decreased with the addition of TPEs. That is, the chain stiffness decreased as a result of weak interactions between TSE and TPE macromolecules. The results of the analysis reveal that, by increasing the TPEs content in the composites, the reduction in *V*_*e*_ due to the attenuate imparted by the polyurethane at high temperatures can be minimized.

## 4. Conclusions

The series of pseudo-semi IPNs with various proportions of TPEs have been synthesized via a sequential process. We take advantage of the high elastic dynamics theory to evaluate the integrity of the network structure. Through the DMA spectra, we acquire the high elasticity modulus, estimating the correlation density parameters.

The elastic modulus of TPEs comes from the contribution of physical crosslinking, which is formed by linear polymer. Additionally, the chemical crosslinking effect could be negligible. The hydrogen bond and physical tangles weaken and diminish following temperature increase. In comparison with those of the TPEs, the pseudo-semi IPNs give a lower modulus due to their interpenetrating network structures. In this sense, low amounts of TPE in semi IPNs (10%) enhance the mobility of pseudo-semi IPNs in comparison with the unattached TSEs, reflected by the position and height of the chemical crosslinking density and physical crosslinking density. This is the result of the lessening/dissociation of the hydrogen bonds between hard segments and soft segments, of which hard segment domains take place during the synthesis of pseudo-semi IPNs in the presence of TPEs. Moreover, the ductility of pseudo-semi IPN increased from 89.6% to 491%, and the strength of the material matrix was significantly improved.

As the temperature increases, the transformations that pseudo-semi IPNs undergo are a strong softening and a defectiveness of the crosslinking, which are discernible in the variations of the viscoelastic parameters. The trend of the *V*_*c*_ and *V*_*p*_ values is consistent with the above statements.

## Figures and Tables

**Table 1:** The parameters of samples, wt.%, stress, strain and glass transition temperature.

Sample	wt.% ^1^	*σ*_m_	*ε*_b_	*T*_g_ (°C)
TPE ^2^	-	3.98	998	−37.68
TSE ^3^	-	0.65	89.6	−80.43
Sample 1	10	0.82	123	−76.51
Sample 2	20	0.94	172.5	−78.17
Sample 3	30	1.13	156.6	−78.17
Sample 4	40	2.44	412.5	−78.18
Sample 5	50	3.32	491.5	−77.68

^1^ The proportions of TPEs at pseudo-semi IPNs. ^2^ The molar ratio of [NCO]/[OH] was 1.0 for TPE. ^3^ The molar ratio of [NCO]/[OH] was 1.3 for TSE.

**Table 2:** The calculating parameters of TPEs (the results are obtained according to Equation (11)).

Sample	*E_a_* (KJ/mol)	*A*	*R*
TPE-1	48.85	2.975 × 10^−12^	0.9984
TPE-2	48.86	3.584 × 10^−12^	0.9947
TPE-3	48.96	2.425 × 10^−12^	0.9997
TPE-4	48.78	4.133 × 10^−12^	0.9998
TPE-5	48.62	3.542 × 10^−12^	0.9999

**Table 3:** Correlation coefficients as well as *V*_*c*_ and *V*_*p*_ constants obtained by experiments and theoretical calculation.

Sample	*E*_a_ (KJ/mol)	*A*	*R*	*V_c_ ×* 10^−4^/(molcm^−3^)	*V_p_* × 10^−4^/(molcm^−3^)
Sample 1	24.41	6.593 × 10^−5^	0.9997	1.92	1.06
Sample 2	27.34	2.661 × 10^−5^	0.9998	1.61	1.37
Sample 3	27.72	6.056 × 10^−5^	0.9997	0.98	1.64
Sample 4	28.40	2.549 × 10^−5^	0.9999	0.95	2.0
Sample 5	31.73	8.797 × 10^−6^	0.9999	0.70	2.58

## Data Availability

The data presented in this study are available upon request from the corresponding author.
